# Low HBV replication does not aggravate disease progression in patients with superinfection with HEV

**DOI:** 10.1038/s41598-025-34028-w

**Published:** 2025-12-27

**Authors:** Li Chen, Sijia Dai, Jianming Wu, Xinyi Gao, Xinyi Liu, Youlin Shao

**Affiliations:** 1 Department of Infectious Diseases, Changzhou Medical Center, Changzhou Wujin People’s Hospital, Nanjing Medical University, 2 Yongning Road, Changzhou City, 213017 Jiangsu China; 2 Department of Hepatology, Changzhou Medical Center, Changzhou Third People’s Hospital, Nanjing Medical University, 300 Lanling Road, Changzhou City, 213001 Jiangsu China; 3 Department of Infectious Diseases, Wujin Hospital Affiliated with Jiangsu University, 2 Yongning Road, Changzhou City, 213017 Jiangsu China; 4 Department of Infectious Diseases, The Wujin Clinical College of Xuzhou Medical University, 2 Yongning Road, Changzhou City, 213017 Jiangsu China

**Keywords:** Hepatitis B virus, Hepatitis E virus, Acute hepatitis E, Superinfection, HBV low viral replication, Disease progression, Retrospective study, Diseases, Gastroenterology, Medical research, Microbiology

## Abstract

In China, co-infection with HEV and HBV is relatively common, and the impact of low HBV DNA load on coinfected patients remains unknown. This study aimed to assess the effect of low-replication hepatitis B virus (HBV) infection on disease progression in patients with hepatitis E virus (HEV) superinfection. This study included 260 consecutive patients diagnosed with sporadic acute hepatitis E (AHE) who were admitted to two hospitals in Jiangsu Province, China, between January 2018 and December 2023. Patients were categorized into two groups: those who were HBsAg-positive with low HBV replication (defined as HBV DNA levels < 2000 IU/mL) and those who were HBsAg-negative. Clinical and laboratory data were retrospectively collected and analyzed to evaluate biochemical parameters, disease progression, and clinical outcomes across the two groups. Of the 260 patients, 24 (9.23%) were HBsAg-positive, while 236 (90.77%) were HBsAg-negative. The average age of participants was 53.48 ± 13.3 years, with no significant differences in age or gender between the two groups. Liver damage manifestations and biochemical indicators were comparable at admission and throughout follow-up. Importantly, there were no significant differences in disease progression or clinical outcomes between the groups. The cumulative rates of liver enzyme and bilirubin normalization were also similar. Although the follow-up period was longer in the HBsAg-positive group (442.79 ± 474.58 days vs. 215.65 ± 346.11 days, *P* = 0.031), no differences in clinical outcomes were identified. In this retrospective cohort, low replication HBV infection did not appear to significantly influence short-term disease progression in patients with HEV superinfection, though larger prospective studies are needed to confirm these findings.

## Introduction

Annually, an estimated 20 million new cases of hepatitis E virus (HEV) infection occur worldwide, with 3.3 million individuals presenting hepatitis symptoms. By 2015, HEV was responsible for 44,000 deaths, representing 3.3% of all fatalities related to viral hepatitis^1^. Recent active surveillance reveals a downward trend in acute hepatitis E (AHE) in China from 2007 to 2017, with an annual average age-standardized incidence rate of 17.50 per 100,000, surpassing the national statutory disease reporting system rate of 10.26 per 100,000^2^. Globally, about 296 million individuals are chronically infected with hepatitis B virus (HBV)^3^. Despite substantial advancements in preventing HBV mother-to-child transmission in China over the past three decades^4^, the prevalence of HBV infection among individuals born before 1992 remains above 5%^5^. In China, the rate of HEV and HBV co-infection has reached as high as 4.99 per 100,000^2^.

Several factors influence the prognosis of patients with AHE. Aging is recognized as a significant risk factor, associated with more severe disease presentations and poorer outcomes in AHE. Research indicates that elderly patients with underlying hepatitis B cirrhosis who experience HEV superinfection exhibit accelerated disease progression and elevated long-term mortality rates among those hospitalized^6^. Additionally, specific clinical parameters, including hepatic encephalopathy, bilirubin levels exceeding 500 µmol/L, severe coagulation abnormalities (international normalized ratio > 2), and a platelet count below 100 × 10^9^/L, are independently linked to higher mortality in AHE patients^7^. Patients with acute type E hepatitis who also have diabetes, cirrhosis, or concurrent hepatitis B infection face a heightened risk of developing liver failure^8^.

Tseng et al. reported a one-year mortality rate of 35.7% in hepatitis B-related cirrhosis patients co-infected with HEV, noting that hepatitis E may accelerate disease progression in chronic hepatitis B patients^9^. Additionally, chronic hepatitis B has been identified as an independent risk factor for increased mortality associated with acute hepatitis E^10^. In comparison to HEV infection alone, HEV loads are elevated in patients with chronic HBV infection who are co-infected with HEV^11^. Moreover, when compared to patients with HBV monoinfection, those with HBV-HEV co-infection exhibit increased expression of cytokines such as IL-6, IL-10, and TNF-α^12^. Furthermore, severe disease manifestations in patients with acute hepatitis E superinfections on chronic hepatitis B have been correlated with cirrhosis and moderate levels of HBV DNA^13^. While one study concluded that HBV treatment does not improve the prognosis of liver failure resulting from AHE superinfection in chronic hepatitis B patients^14^, another study found that successful antiviral therapy may mitigate the adverse effects of HBV DNA replication in liver cirrhosis patients with AHE superinfection on chronic hepatitis B^13^.

Prior research has predominantly concentrated on the effects of HBV superinfection on AHE, yet a deeper understanding of the relationship between HBV DNA viral load and HEV superinfection is vital for enhancing clinical practice, especially in regions with high co-infection rates, such as China. Patients with non-replicating chronic HBV infection or those receiving effective antiviral therapy may exhibit undetectable HBV DNA or low viral loads^15^. However, most previous studies have not stratified CHB patients by HBV DNA levels, and the specific impact of low-level viremia (e.g., < 2000 IU/mL) on the outcome of HEV superinfection remains unclear. Clarifying this issue is clinically relevant for risk stratification and management. The prognostic significance of low viral load chronic HBV infection in individuals superinfected with HEV is yet to be established. Therefore, this study aims to examine the clinical and prognostic outcomes of superinfection with low viral load chronic HBV in acute HEV-infected individuals.

## Methods

### Patient selection

This study, conducted from January 2018 to December 2023, involved consecutive hospitalized patients with acute hepatitis E (AHE) at Wujin People’s Hospital and the Third People’s Hospital in Changzhou, Jiangsu Province, China. Consecutive inpatient AHE cases were classified into two groups based on HBV infection status: HBsAg-positive patients with low HBV replication (assessed by real-time fluorescent quantitative PCR, defined as HBV DNA levels < 2000 IU/mL) and HBsAg-negative patients. All participants were confirmed to be free from hepatitis A, C, and D virus infections and had no prior diagnoses of autoimmune liver disease or decompensated cirrhosis. Data for this study were derived solely from clinical medical records without any additional blood sampling from the patients. The study, approved by the Ethics Committee of Wujin People’s Hospital in Changzhou City (IRB-GL1-AF06), was conducted in accordance with the principles, guidelines and recommendations provided by the ethics committee.

Given the retrospective nature of this study, which involved no patient identification, privacy disclosure, or direct risk, informed consent was waived by the Ethics Committee of Wujin People’s Hospital of Changzhou.

Acute hepatitis E is characterized by the presence of positive HEV immunoglobulin M and immunoglobulin G antibodies in patients exhibiting acute hepatitis symptoms along with liver dysfunction^16^. Chronic HBV infection is identified in individuals who have been HBsAg positive for six months or longer. Low viral load chronic HBV infection is defined by HBsAg positivity and HBV DNA levels below 2000 IU/mL, irrespective of antiviral treatment status^15^. AHE superinfections on chronic hepatitis B (CHB) refer to cases of acute HEV infection occurring in patients with pre-existing chronic HBV infection. Metabolic-associated fatty liver disease (MAFLD) is defined according to the guidelines of the 2020 International Expert Group on MAFLD terminology^17^. Heavy drinking is specified as alcohol consumption of ≥ 30 g/day for men and ≥ 20 g/day for women. Diabetes mellitus is defined according to the guideline for the prevention and treatment of type 2 diabetes mellitus in China (2020 edition)^18^.

### Sample collection and testing

Clinical and laboratory biochemical, hematological, serological, and imaging data were extracted from medical records. Commercially available enzyme-linked immunosorbent assay kits were employed to detect HEV immunoglobulin M and immunoglobulin G antibodies in serum samples.

### Statistical analysis

Categorical variables were presented as frequency and percentage, with inter-group differences assessed using the chi-square test or Fisher’s exact test. Continuous variables were generally described as mean ± standard deviation. The Kolmogorov-Smirnov test was applied to evaluate normality, while the Levene test assessed homogeneity of variance. For continuous variables meeting both normal distribution and homogeneity of variance assumptions, inter-group differences were evaluated using the *t*-test; for variables not following a normal distribution, the Mann-Whitney U non-parametric test was applied. The Kaplan-Meier method was utilized to analyze and plot the cumulative recurrence rate. Statistical significance was defined as a *P*-value less than 0.05. To evaluate the robustness of the primary clinical outcome given the small sample size of the HBsAg-positive group, a non-parametric bootstrap resampling method (10,000 replicates) was employed to calculate the 95% confidence interval (CI) for the inter-group risk difference (HBsAg-positive minus HBsAg-negative) in liver failure incidence. All analyses and visualizations were conducted using R.

## Results

### Epidemiological and demographic characteristics of patients

**Fig. 1:**
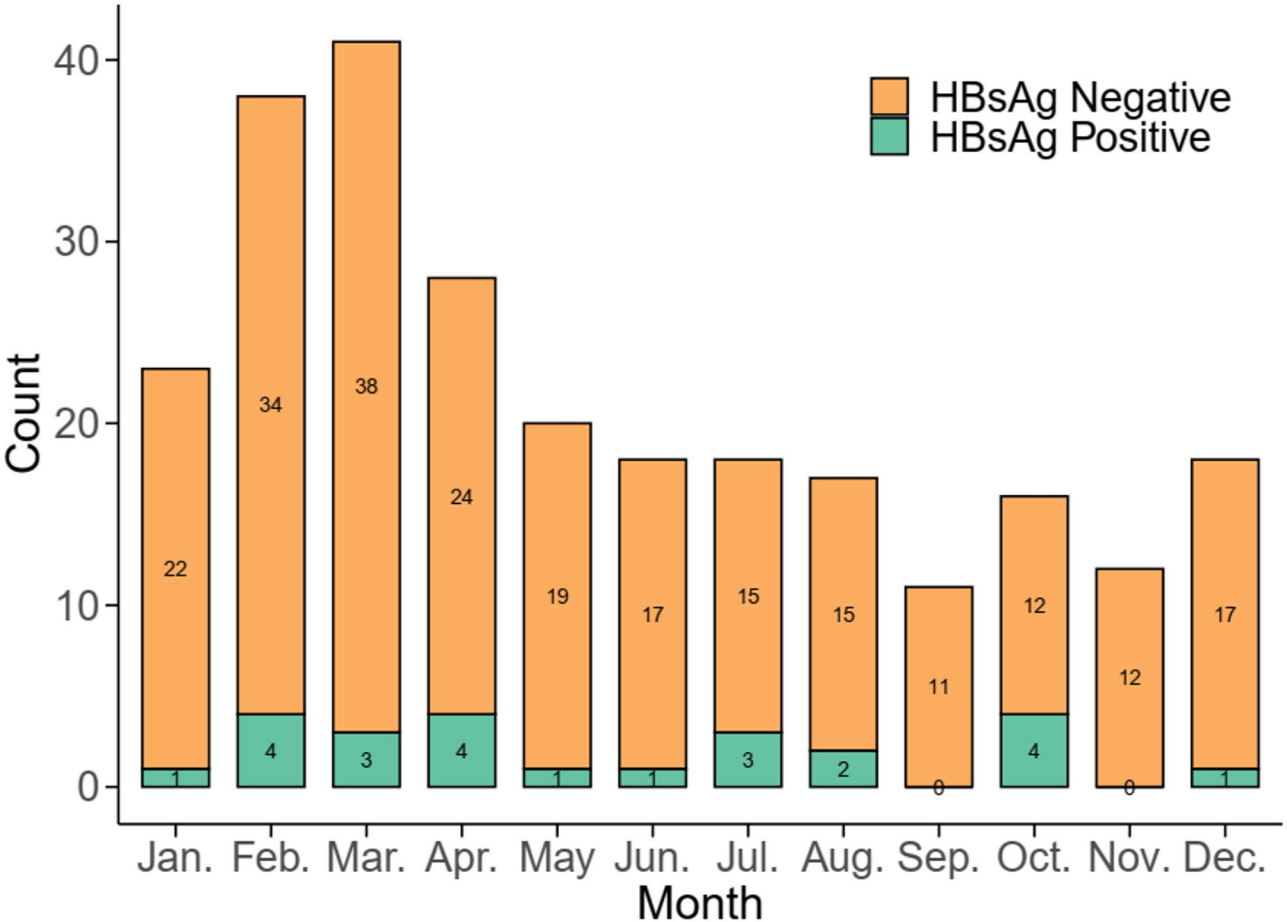
Monthly distribution of acute hepatitis E cases from 2018 to 2023 (*N* = 260).

**Fig. 2:**
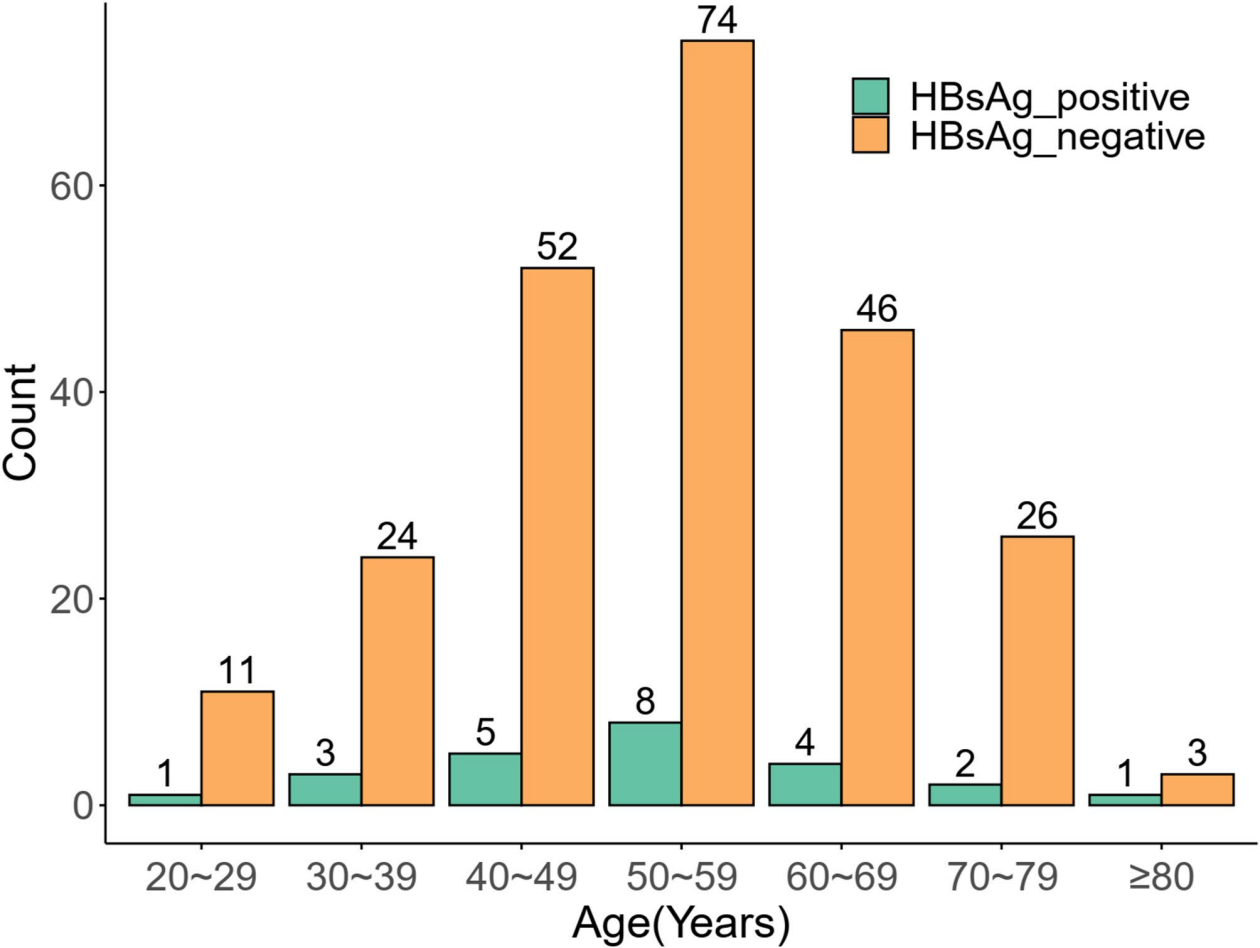
Age distribution of acute hepatitis E cases from 2018 to 2023 (*N* = 260).

A total of 260 patients diagnosed with AHE were included in the study, comprising 24 cases (9.23%) in the HBsAg-positive group and 236 cases (90.77%) in the HBsAg-negative group. Among the 24 HBsAg-positive patients with low HBV replication, 8 (33.3%) were receiving ongoing oral nucleos(t)ide analogue therapy at the time of acute HEV superinfection: six were on entecavir and two on tenofovir disoproxil fumarate. The median duration of antiviral therapy was 7 years (range, 2–18 years). The remaining 16 patients (66.7%) were not under any antiviral treatment prior to admission. AHE cases occur sporadically throughout the year, with peak incidence observed in February, March, and April. As shown in Fig. 1, the difference in incidence between the two groups was not statistically significant, with a *P*-value of 0.24. The onset age of AHE ranges from 20 to 88 years, with a peak between 40 and 69. Figure 2 compares age distributions across the HBsAg-positive and -negative groups, indicating a *P*-value of 0.95. The baseline characteristics of the study population are summarized in Table 1. The two groups were well-balanced in terms of age, sex, BMI, and major comorbidities, although the HBsAg-positive group was relatively small (*n* = 24).

**Table 1:** Demographic characteristics of acute hepatitis E cases from 2018 to 2023.

Variables	Total	HBsAg-positive	HBsAg-negative	χ²/t	*P*
Age(years)	53.48 ± 13.3	52.54 ± 13.71	53.57 ± 13.28	258	0.718
Sex(male)	181(69.62%)	15(62.5%)	166(70.34%)	0.633	0.426
BMI	23.25 ± 3.17	23.4 ± 2.77	23.24 ± 3.21	-0.241	0.810
Nutritional status					
Obesity	64(24.62%)	7(29.17%)	57(24.15%)	1.462	0.691
Overweight	69(26.54%)	7(29.17%)	62(26.27%)		
Normal weight	116(44.62%)	10(41.67%)	106(44.92%)		
Emaciation	11(4.23%)	0(0)	11(4.66%)		
MAFLD	85(32.69%)	6(25%)	79(33.47%)	0.711	0.399
Heavy drinking	43(16.54%)	2(8.33%)	41(17.37%)	0.718	0.397
Smoking	31(11.92%)	1(4.17%)	30(12.71%)	0.810	0.368
Hypertension	69(26.54%)	6(25%)	63(26.69%)	0.032	0.858
Diabetes	28(10.77%)	1(4.17%)	27(11.86%)	0.562	0.453
Tumor*	11(4.23%)	1(4.17%)	10(4.24%)	5.585e-32	0.731
Lung disease	25(9.62%)	1(4.17%)	24(10.17%)	0.345	0.557
Thyroid disease	14(5.38%)	0(0%)	14(5.93%)	0.566	0.452
Blood system	14(5.38%)	0(0%)	14(5.93%)	0.566	0.452
Autoantibody	50(19.23%)	7(29.17%)	43(18.22%)	1.681	0.306
ANA positive**	40(15.38%)	6(25%)	34(14.41%)	1.878	0.283

Tumor*: history of any malignancy; Autoantibody**: positivity for any non-organ-specific autoantibody.

### Clinical Manifestations and Treatment of Patients

Patients sought medical attention an average of 10.25 ± 8.85 days following symptom onset. The most frequently reported symptoms were fatigue, reduced appetite, and dark urine, with incidence rates of 87.31%, 83.08%, and 83.85%, respectively. Additionally, 32.31% of patients experienced a transient fever, typically low-grade. Nausea and vomiting were observed in 26.92% and 13.08% of patients, respectively, while diarrhea and abdominal pain were less frequently reported. Besides standard treatments such as glycyrrhetinic acid preparations, polyene phosphatidylcholine, s-adenosylmethionine, and ursodeoxycholic acid, 12.50% of HBsAg-positive patients and 19.07% of HBsAg-negative patients received at least one dose of glucocorticoid therapy, with a *P*-value of 0.607, as shown in Table 2.

**Table 2:** Clinical manifestations of acute hepatitis E from 2018 to 2023.

Variables	Total	HBsAg positive	HBsAg negative	*P*
Chief complaint days	10.25 ± 8.85	9.46 ± 6.74	10.33 ± 9.04	0.645
Fatigue	227(87.31%)	19(79.17%)	208(88.14%)	0.349
Loss of appetite	216(83.08%)	19(79.17%)	197(83.47%)	0.802
Dark urine	218(83.85%)	19(79.17%)	199(84.32%)	0.717
Fever	84(32.31%)	7(29.17%)	77(32.63%)	0.73
Nausea	70(26.92%)	4(16.67%)	66(27.97%)	0.234
Vomiting	34(13.08%)	2(8.33%)	32(13.56%)	0.685
Diarrhea	5(1.92%)	1(4.17%)	4(1.69%)	0.952
Abdominal pain	4(1.54%)	0(0%)	4(1.69%)	0.309
Liver failure	13(5.00%)	2(8.33%)	11(4.66%)	0.768

Comparison of Initial Biochemical Parameters upon Hospital Admission between HBsAg-Positive and HBsAg-Negative Patient Groups.

At hospital admission, no statistically significant differences were detected in markers of liver cell injury (ALT and AST), indicators of bile stasis (ALP, GGT, and TBA), liver synthesis function parameters (ALB, PA, PT, and CHE), or renal function measures (BUN and CREA) between the two groups of patients. Nevertheless, as indicated in Table 3, statistically significant differences were present in IgG levels, spleen thickness, white blood cell counts, and platelet counts.

**Table 3:** Comparison of initial biochemical parameters between HBsAg-positive and HBsAg-negative Groups.

Variables	Total	HBsAg-positive	HBsAg-negative	*P*
ALT (IU/L)	1273.5 ± 1075.08	1365.91 ± 1055.19	1264.1 ± 1078.84	0.659
AST (IU/L)	758.96 ± 944.21	828.17 ± 868.42	751.93 ± 953.02	0.707
ALP (IU/L)	199.72 ± 85.84	190.96 ± 59.19	200.62 ± 88.18	0.600
GGT (IU/L)	262.17 ± 217.27	205.33 ± 188.62	268 ± 219.53	0.179
TBA (µmol/L)	141.22 ± 125.49	124.54 ± 104.93	142.98 ± 127.54	0.495
TBIL (µmol/L)	101.72 ± 92.01	92.68 ± 78.4	102.64 ± 93.38	0.614
DBIL (µmol/L)	75.04 ± 68.62	70.97 ± 63.35	75.45 ± 69.24	0.766
ALB (g/L)	36.91 ± 5.13	37.24 ± 4.92	36.87 ± 5.16	0.737
GLO (g/L)	28.64 ± 4.74	30.08 ± 4.81	28.49 ± 4.72	0.119
PA (mg/dL)	12.31 ± 6.99	9.95 ± 3.33	12.56 ± 7.23	0.081
CHE (IU/L)	5930.11 ± 1881.51	5603.42 ± 2082.31	5964.35 ± 1860.89	0.372
CRP (IU/L)	9.45 ± 12.03	7.44 ± 7.52	9.67 ± 12.41	0.388
BUN (mmol/L)	5.04 ± 6.25	4.7 ± 1.64	5.07 ± 6.55	0.784
CREA (µmol/L)	67.01 ± 24.44	64.96 ± 9.2	67.23 ± 25.52	0.666
IgM (g/L)	2.23 ± 1.12	2.38 ± 1.28	2.21 ± 1.1	0.535
IgA (g/L)	2.66 ± 1.12	2.62 ± 0.85	2.66 ± 1.15	0.887
IgG (g/L)	14.93 ± 4.07	16.91 ± 3.8	14.72 ± 4.05	**0.022**
PT (s)	14.05 ± 1.93	14.53 ± 1.74	14.01 ± 1.95	0.219
PTA (%)	87.62 ± 20.8	80.12 ± 17.12	88.36 ± 21.01	0.070
INR	1.06 ± 0.19	1.1 ± 0.17	1.05 ± 0.19	0.240
WBC (×E + 09/L)	5.63 ± 2.15	4.49 ± 1.81	5.75 ± 2.15	**0.006**
RBC (g/L)	4.53 ± 0.6	4.42 ± 0.51	4.54 ± 0.6	0.325
PLT (×E + 09/L)	183.71 ± 68.18	153.17 ± 64.91	186.81 ± 67.86	**0.021**
Spleen length (cm)	103.08 ± 33.07	110.69 ± 20.16	102.29 ± 34.07	0.237
Spleen thickness (cm)	38.13 ± 6.89	41.77 ± 7.97	37.73 ± 6.66	**0.006**

Comparison of Liver Function Recovery and Disease Progression between HBsAg-Positive and HBsAg-Negative Patient Groups.

**Fig. 3:**
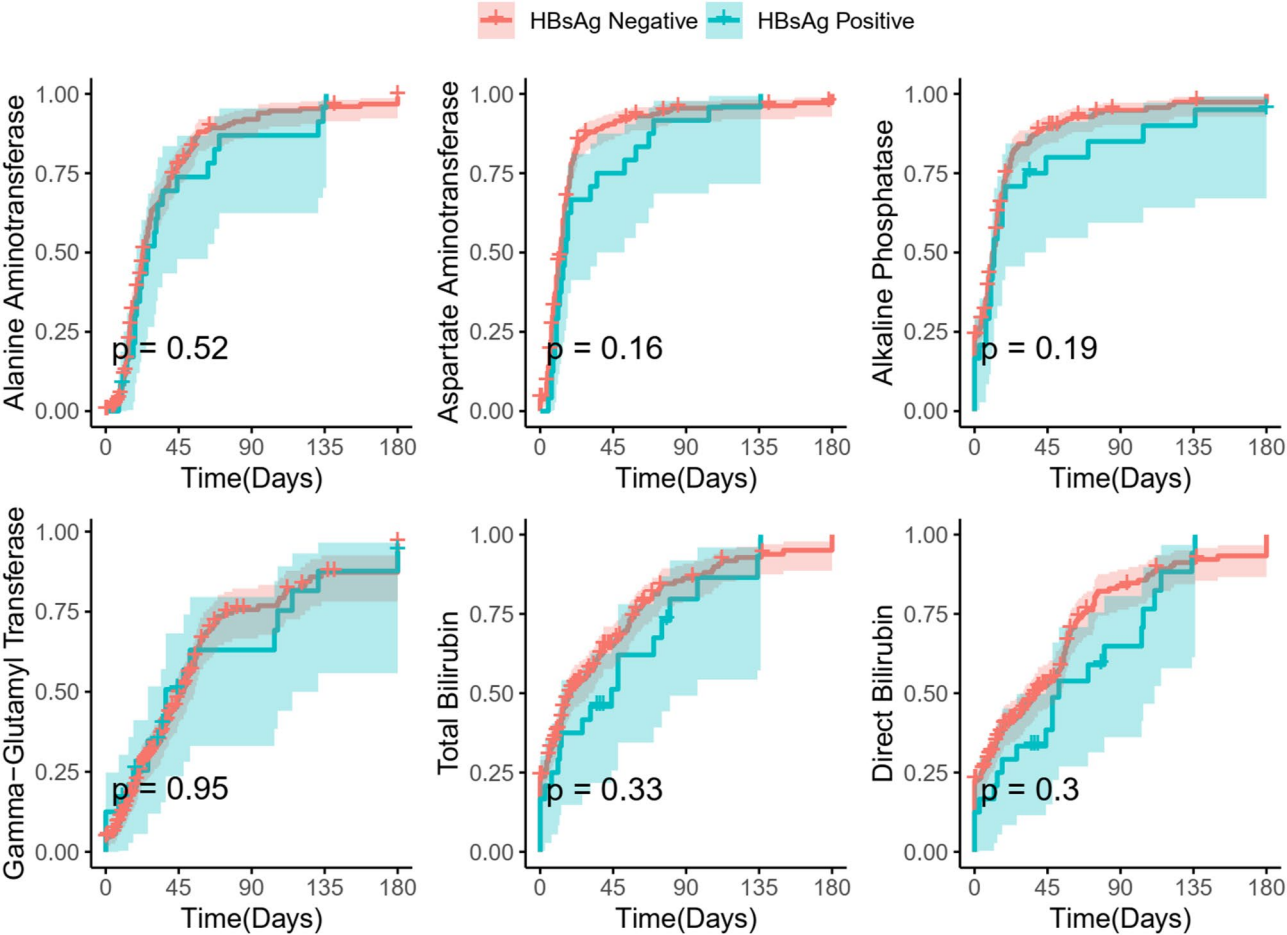
Cumulative normalization rates of Alanine Aminotransferase, Aspartate Aminotransferase, Alkaline Phosphatase, Gamma-glutamyl Transferase, Total Bilirubin, and Direct Bilirubin in AHE patients.

Throughout the disease course, 13 patients (5.00%) developed liver failure without progressing to hepatic encephalopathy, hepatorenal syndrome, or mortality. No statistically significant differences were identified in disease progression or clinical outcomes between the two patient groups. Bootstrap sensitivity analysis confirmed the robustness of this finding. The 95% confidence interval for the risk difference in liver failure incidence (HBsAg-positive vs. HBsAg-negative) was − 5.93% to + 16.60%, which includes zero. By the final assessment, the proportions of patients with ALT, AST, ALP, GGT, TBIL, DBIL, and IBIL levels below twice the upper limit of normal were 93.46%, 98.46%, 99.23%, 64.23%, 95.77%, 56.54%, and 98.85%, respectively. The distribution of these key indicators at the final assessment did not exhibit statistically significant differences between the groups (Table 4). Likewise, no significant variation was found in the normalization rate of major liver function indicators between the two cohorts (Fig. 3). Patient follow-up durations were (442.79 ± 474.58) days and (215.65 ± 346.11) days, respectively, with a *P*-value of 0.0026. The extended follow-up in the HBsAg-positive group likely reflects the chronic nature of HBV infection requiring ongoing monitoring, rather than a difference in the acute HEV disease course.

**Table 4:** Distribution of the status of main liver function indicators in the last examination during Hospitalization, N (%).

Variables status	ALT		AST		ALP		GGT		TBIL		DBIL		IBIL	
	HBsAg-negative	HBsAg-positive	HBsAg-negative	HBsAg-positive	HBsAg-negative	HBsAg-positive	HBsAg-negative	HBsAg-positive	HBsAg-negative	HBsAg-positive	HBsAg-negative	HBsAg-positive	HBsAg-negative	HBsAg-positive
<1×ULN	173(73.31)	21(87.5)	211(89.41)	21(87.5)	195(82.63)	19(79.17)	60(25.42)	9(37.5)	138(58.47)	13(54.17)	73(30.93)	7(29.17)	225(95.34)	24(100)
1~<2×ULN	47(19.92)	2(8.33)	20(8.47)	3(12.5)	39(16.53)	5(20.83)	89(37.71)	9(37.5)	87(36.86)	11(45.83)	61(25.85)	6(25)	8(3.39)	0(0)
2~<3×ULN	7(2.97)	1(4.17)	2(0.85)	0(0)	2(0.85)	0(0)	42(17.8)	3(12.5)	3(1.27)	0(0)	40(16.95)	7(29.17)	0(0)	0(0)
≥ 3×ULN	9(3.81)	0(0)	3(1.27)	0(0)	0(0)	0(0)	45(19.07)	3(12.5)	8(3.39)	0(0)	62(26.27)	4(16.67)	3(1.27)	0(0)
*P*	0.4032		0.6704		0.6493		0.6059		0.7399		0.4837		1	

## Discussion

The objective of this study is to examine the influence of chronic HBV infection with a low viral load on the clinical progression and prognosis of patients experiencing HEV superinfection. A retrospective analysis of 260 patients with AHE revealed that low viral load HBV infection does not significantly worsen disease progression in patients with HEV superinfection. These findings offer valuable insights into the clinical management of such superinfections.

From a global epidemiological perspective, co-infection with HBV and HEV is relatively common, particularly in regions with high HBV prevalence^2,19^. Research on the effect of HEV superinfection on HBV DNA viral load remains limited, with contradictory findings. These discrepancies may be attributed to variations in study populations, including differences in HBV replication status, degree of liver fibrosis, and comorbidities. Hoan et al. reported that HBV DNA levels in CHB patients with acute HEV superinfection were higher than those in patients with prior HEV infection or without HEV^20^. Conversely, other studies found no significant difference in HBV DNA levels between CHB patients with HEV superinfection and those with only CHB^21^, with some indicating even lower HBV DNA levels in both serum and liver tissue^12^. Despite the ongoing uncertainty regarding HEV superinfection’s influence on HBV replication, it is widely accepted that a lower HBV DNA load has a minimal impact on liver disease progression^15,22^. Our study specifically focused on CHB patients with low-level viremia (HBV DNA < 2000 IU/mL), a subgroup that has received less attention in previous research. We found that these patients had similar short-term outcomes compared to HBsAg-negative individuals following HEV superinfection. In our cohort, the low HBV DNA load could be attributed to either long-term effective antiviral suppression (33.3% of patients were on therapy, predominantly with entecavir or tenofovir, for a median duration of 7 years) or a relatively inactive infection state in treatment-naïve individuals. Crucially, irrespective of the mechanism—pharmacological or immunological—this low-replication state did not predispose patients to more severe HEV disease. This observation suggests that the detrimental effects of HEV superinfection are likely more pronounced in patients with higher HBV DNA levels or advanced underlying liver disease, such as cirrhosis. The mechanisms underlying the interaction between low-replication HBV and HEV require further elucidation. Viral interference, wherein one virus modulates the replication of another, is a plausible explanation. In vitro studies suggest HEV and HBV may influence each other’s replication cycles^11^. Direct competition for common cellular entry receptors is less likely, as HBV entry is mediated primarily by NTCP^23^, whereas HEV utilizes a distinct, multi-receptor process^24^. In our cohort, the low HBV viral load and antigen production may have been insufficient to substantially alter the intrahepatic immune milieu or compete for cellular resources with HEV. This interpretation aligns with the broader understanding that HEV infection outcomes are shaped by a combination of viral features and the host’s immune status^25^; in our patients’ specific virological context, HEV infection did not appear to synergize with low-load HBV to trigger aberrant immune responses or worsen outcomes. In summary, our findings indicate that in individuals with well-controlled or inactive CHB, HEV superinfection may follow a self-limited course similar to that in HBsAg-negative patients. Future studies using advanced models are warranted to dissect these virological and immunological interactions at the molecular level.

This study found that, despite co-infection with HBV and HEV, patients with low-level HBV viremia demonstrated liver function recovery, disease progression, and clinical outcomes similar to those of AHE patients without HBV infection, within the context of our study sample. This result contrasts with some previous studies, which frequently suggest that HBV infection, particularly in patients with liver cirrhosis, accelerates disease progression in HEV-infected individuals^8,9^. However, these earlier studies did not specifically examine HBV DNA viral load, whereas this investigation focused on patients with low viral loads, thereby highlighting the distinct impacts of HBV on HEV infection prognosis depending on the HBV infection state. The similar rates of liver failure and biochemical normalization observed in both groups further support the notion that a low HBV viral load may not additively worsen the acute hepatic insult caused by HEV.

Consistent with the clinical outcomes, serial liver function parameters, including ALT, AST, and TBIL, showed no significant differences between the two groups during hospitalization and recovery. This reinforces the biochemical parallel to the comparable clinical course. These findings support the hypothesis that low-replication HBV infection has a limited or potentially minimal prognostic impact on patients with HEV superinfection.

Notably, the follow-up time of patients in the HBsAg-positive group was significantly longer than that of patients in the HBsAg-negative group. This result is related mainly to patients’ understanding of the chronicity of HBV infection and their education in HBV-infected individuals. It may also be possible that during long-term follow-up, low viral load HBV infection may still have a certain impact on patients’ liver health, but this impact has not yet been demonstrated in the current research cycle. Our study has several limitations that should be considered when interpreting the results. First, the retrospective design inherently carries risks of selection and information bias. Second, and most importantly, the number of HBsAg-positive patients with low HBV replication was small (*n* = 24), which limits the statistical power to detect subtle differences in outcomes and precludes meaningful multivariate adjustment for potential confounders. Although the bootstrap sensitivity analysis supported the primary conclusion (95% CI for risk difference included zero), the wide confidence interval (-5.93% to + 16.60%) underscores the imprecision of the estimate due to the limited sample size. This sample size reflects the challenge of enrolling this specific patient subgroup but necessitates cautious interpretation and confirmation in larger studies. Third, data on several potentially relevant variables were unavailable, including HBeAg status, anti-HBc for HBsAg-negative patients (to rule out occult HBV), HEV RNA levels, and precise quantification of liver fibrosis via elastography or biopsy. Although indirect non-invasive scores like FIB-4 (Fibrosis-4 index) and APRI (aspartate aminotransferase-to-platelet ratio index) are widely used in chronic liver disease assessment, their application during acute hepatitis is problematic. During acute HEV infection, markedly elevated transaminases artificially inflate these scores, leading to potential overestimation of fibrosis. Therefore, we did not rely on these indices to characterize baseline liver injury status in our analysis. The significantly longer follow-up duration in the HBsAg-positive group, while likely related to standard chronic disease management, could also introduce surveillance bias. Furthermore, although key comorbidities like heavy drinking, MAFLD and diabetes were balanced between groups, we cannot fully exclude residual confounding. Future prospective studies with larger sample sizes, systematic virological and fibrosis assessment, and standardized follow-up are needed to validate our findings and explore long-term outcomes. Therefore, future research should consider extending the follow-up time to evaluate the long-term prognosis of patients with AHE superinfections in CHB patients more comprehensively.

This study also observed that patients in the HBsAg-positive group exhibited higher IgG levels, increased spleen thickness, and lower counts of white blood cells and platelets. Splenomegaly^26^, leukopenia, and thrombocytopenia^27^ are commonly observed in chronic HBV-infected patients, even among those with normal or slightly elevated alanine aminotransferase levels. These variations may suggest subtle impacts of chronic HBV infection on the host’s immune status and disease progression. Although patients with CHB lack HBV-specific antibodies, their serum levels of total IgG are significantly higher than those of healthy controls. This phenomenon is likely related to the activation of systemic peripheral B cells induced by HBV infection, coupled with a decrease in the function of B cells that secrete anti-HBs^28^. Nevertheless, in alignment with the primary findings, these immune differences did not result in significant clinical prognostic disparities. This suggests that, under low viral load conditions, HBV’s influence on the host immune system is likely limited and gradual, with the short duration potentially insufficient to markedly impact disease progression in HEV-infected patients with superinfection.

Recent literature indicates that research on AHE superinfections in CHB patients is advancing significantly. Studies highlight that factors such as advanced age^6^, coexisting cirrhosis, and diabetes^7^ can elevate the risk of liver failure in HEV-infected individuals. While some research suggests that antiviral therapy for CHB may not improve the prognosis of liver failure caused by superinfections^14^, other studies demonstrate that antiviral treatment can markedly enhance outcomes in patients with HBV infection and related cirrhosis. This study revealed that low HBV replication does not aggravate disease progression in patients with superinfection with HEV. Effective antiviral therapy has the potential to counteract the adverse effects of HBV DNA replication^13^. All of these findings underscore the importance of keeping HBV DNA levels low to facilitate recovery in CHB patients with AHE superinfections. Consequently, for patients experiencing HBV replication, initiating anti-HBV treatment promptly is advisable.

From a clinical standpoint, monitoring HBV viral load status is crucial in patients with AHE superinfections on CHB. Our findings reveal that patients with low HBV viral loads can be primarily managed for HEV infection, as the influence of HBV on disease outcomes seems minimal. However, for Hepatitis B-related cirrhosis patients with low HBV viral load, the current consensus^29^ indicates that antiviral therapy should be actively considered to mitigate the potential impact of HBV replication on disease progression. This underscores the importance of tailored treatment plans based on individual patient characteristics. For those with low-level viremia but no evidence of advanced fibrosis, our data suggest that management can focus on the acute HEV infection, while maintaining monitoring of HBV status per relevant guidelines.

It is important to acknowledge the limitations of our retrospective study, including its limited sample size. Larger prospective studies are warranted to validate our findings and enhance our understanding of the interplay between HBV and HEV in CHB patients. Additionally, our study noted a significantly longer follow-up time in the HBsAg-positive group, potentially reflecting patients’ awareness of chronic HBV infection. However, the long-term impact of low HBV viral load on liver health in AHE patients remains unexplored within our study period, necessitating extended follow-up in future research. Furthermore, although our discussion mentioned age, coexisting cirrhosis, and diabetes as potential risk factors for hepatic failure in HEV-infected individuals, their interaction with HBV viral load and influence on study results were not fully explored. Future studies should control for these variables to more accurately assess the impact of HBV viral load on prognosis.

## Conclusion

In conclusion, this study provides a preliminary indication that in patients with chronic HBV infection and low-level viremia, HEV superinfection may not lead to a more severe short-term disease course compared to HEV mono-infection. These findings offer a valuable nuanced perspective for clinical management. However, the retrospective design and small sample size of the co-infected group necessitate caution, and further large-scale, prospective research is required to confirm these observations and investigate potential long-term effects.

## Data Availability

The data used to support the findings of this study are available from the corresponding author upon request.

## Abbreviations

AHE: Acute hepatitis E
ALB: Albumin
ALP: Alkaline phosphatase
ALT: Alanine aminotransferase
ANA: Antinuclear antibody
APRI: Aspartate aminotransferase-to-platelet ratio index
AST: Aspartate aminotransferase
BMI: Body mass index
BUN: Blood urea nitrogen
CHB: Chronic hepatitis B
CHE: Cholinesterase
CREA: Creatinine
CRP: C-reactive protein
DBIL: Direct bilirubin
DNA: Deoxyribonucleic acid
FIB-4: Fibrosis-4 index
GGT: Gamma-glutamyl transferase
GLO: Globulin
HBV: Hepatitis B virus
HEV: Hepatitis E virus
IgA: Immunoglobulin A
IgG: Immunoglobulin G
IgM: Immunoglobulin M
INR: International normalized ratio
MAFLD: Metabolic-associated fatty liver disease
PA: Prealbumin
PCR: Polymerase chain reaction
PLT: Platelet
PT: Prothrombin time
PTA: Prothrombin time activity
RBC: Red blood cell
TBA: Total bile acids
TBIL: Total bilirubin
WBC: White blood cell
